# Situs inversus abdominalis, polysplenia, complex jejunal atresia and malrotation in a neonate: A rare association

**DOI:** 10.1016/j.ijscr.2019.02.016

**Published:** 2019-02-20

**Authors:** Abhishek Chinya, Kirti Naranje, Ankur Mandelia

**Affiliations:** a Department of Pediatric Surgery, Sanjay Gandhi Post Graduate Institute of Medical Sciences, Lucknow, India; b Department of Neonatology, Sanjay Gandhi Post Graduate Institute of Medical Sciences, Lucknow, India

**Keywords:** Situs inversus, Polysplenia, Complex jejunal atresia, Apple-peel atresia

## Abstract

**Introduction:**

Situs inversus, polysplenia, complex jejuna atresia are rare anomalies in isolation. Their association in a single patient is even rarer with challenges in diagnosis and management.

**Presentation of case:**

A 5 day old neonate presented with features of small bowel obstruction. Radiological investigations revealed situs inversus abdominalis with dilated proximal small bowel loops. At laparotomy, abdominal situs inversus, polysplenia, multiple jejunal atresias with apple peel appearance of the ileum with malrotation was seen.

**Conclusions:**

The association of situs inversus, polysplenia and complex jejunal atresia is very rare. Pre-operative diagnosis of situs inversus is important for appropriate incision placement and surgical planning.

## Highlights

• A rare association of situs inversus, polysplenia and complex jejunal atresia is presented here.
• Cardiac and gastro-intestinal anomalies shouls always be suspected and investigated in a child with situs inversus.
• Pre-operative diagnosis of situs inversus has important implications for incision placement and surgical planning.

## 1 Introduction

Situs inversus is a rare anomaly in which the body organs are mirrored from their normal position [1]. It is known to be commonly associated with cardiac and gastro-intestinal anomalies requiring surgical correction. Pre-operative diagnosis of situs inversus is necessary for appropriate incision placement and judgement during surgery. Polysplenia is defined as presence of more than one spleens. It is associated with situs inversus in about 20% of the cases [2]. The incidence of jejuno-ileal atresias is about 1 in 5000 live births [3]. The association of these three conditions is very rare. The authors report that this work has been reported in line with the SCARE criteria [4]. Informed consent was obtained from parents for reporting this case.

## 2 Case report

**Fig. 1:**
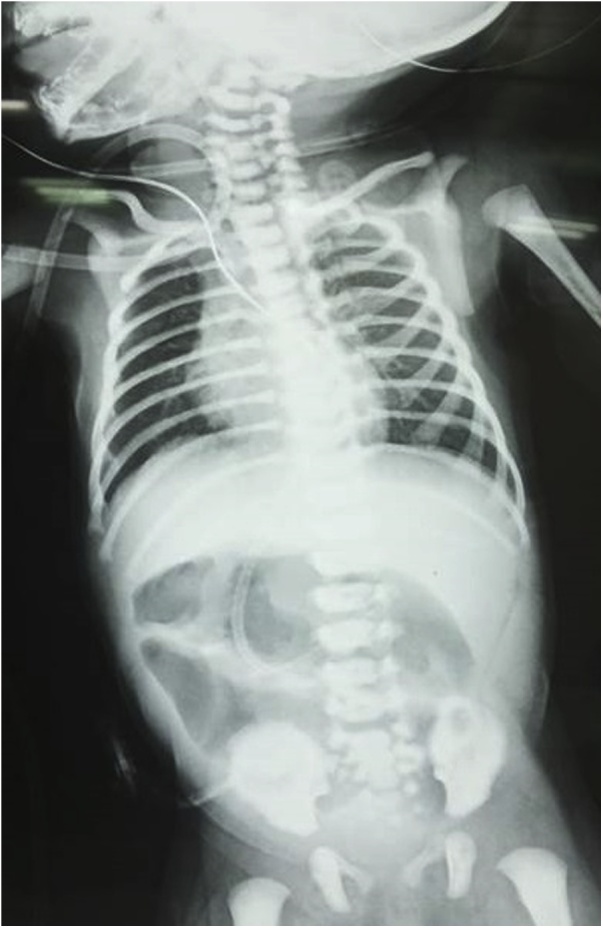
X-ray abdomen showing few dilated bowel loops, paucity of distal gas shadows and nasogastric tube in the right upper quadrant of abdomen.

A 5-day-old girl was referred to us with bilious vomiting and abdominal distention since birth. The baby was born at term by caesarean section to a 30 year old primigravida mother. The baby weighed 2.79 kg at birth. Antenatal ultrasonography at 32 weeks gestation had revealed multiple, dilated, fluid filled bowel loops. At admission, the baby was dehydrated with a distended abdomen. Rest of the systemic examination was unremarkable. Blood investigations were within normal range. X-ray abdomen revealed few dilated bowel loops, paucity of distal gas shadows and nasogastric tube in the right upper quadrant of abdomen (Fig. 1). Ultrasound abdomen revealed liver predominantly on the left side of abdomen, multiple spleen like structures on the right side with distended bowel loops. Echocardiography revealed ostium secundum atrial septal defect of 5 mm with levocardia.

**Fig. 2:**
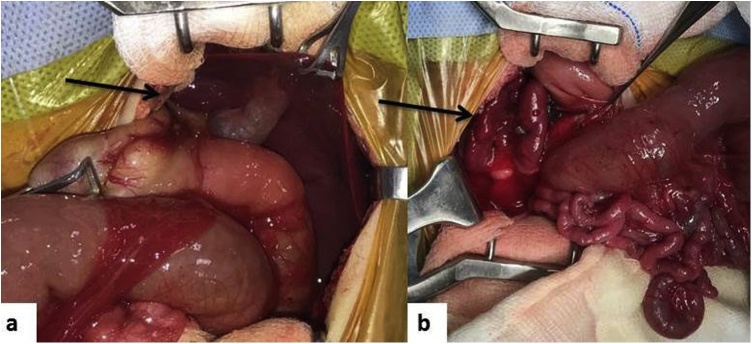
(a) Laparotomy showing liver and duodenal C loop on the left side (arrow), (b) Three spleens on the right side (arrow).

**Fig. 3:**
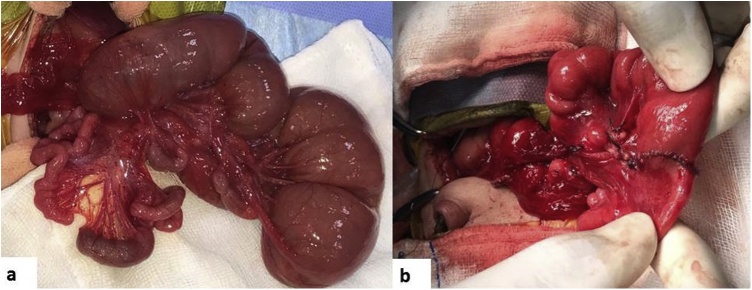
(a) Intra-operative photograph showing massively dilated proximal jejunum with multiple atresias, (b) Completed jejuno-ileal anastomosis.

Since pre-operative diagnosis of abdominal situs inversus was known, a left upper quadrant transverse incision was given in contrast to the classical right upper quadrant transverse incision. Laparotomy confirmed situs inversus abdominis with the liver and duodenal C loop on the left side (Fig. 2a). The baby had three spleens on the right side (Fig. 2b). The proximal jejunum was massively dilated and there were multiple jejunal atresias (5 in number) starting from about 45 cm from the duodenojejunal flexure (Fig. 3a). The entire distal small bowel was supplied by a single branch of the ileo-colic artery and had apple peel appearance. There were several Ladd’s bands crossing the second part of duodenum with narrow base mesentery. The segment of jejunum containing the atretic segments was excised and cut back of the bulbous proximal jejunal end was done. End to side jejuno-ileal anastomosis was done (Fig. 3b), Ladd’s procedure done and a trans-gastric trans-anastomotic feeding jejunostomy done. The residual small bowel length was about 145 cm.

Post-operatively the child was shifted to newborn ICU. Immediate post-op period was uneventful. The baby was started on tube feeds by post-op day (POD) 3, however oral feeds were not tolerated till POD 14. This may be due to hypo-peristalsis in the dilated proximal jejunum. Full oral feeds were reached by POD 28 and the baby was discharged. At 18 months follow up, the baby is thriving well and is asymptomatic.

## 3 Discussion

The normal anatomy of internal organs is known as situs solitus. Mirror transposition of situs solitus is known as situs inversus wherein the solid organs are mirrored. It can be either total or partial. Situs inversus has been known to be associated with multiple anomalies – the most common being cardiac and abdominal. Fonkalsrud et al., in their study of patients with situs inversus, showed 15 patients with situs inversus having major abdominal anomalies requiring surgical attention and 8 of them had duodenal or jejunal atresias [5]. The diagnosis of situs inversus preoperatively is important in planning surgical incision and abdominal procedures.

Polysplenia refers to presence of two or more spleens. The location and number of spleens are variable; their number may range from 2 to 16. The common presentation is vague abdominal pain, nausea, and vomiting. Polysplenia syndrome refers to its association with various organ abnormalities in the abdomen and chest. It is often recognized in childhood, though about 10% cases present late during adulthood. Situs inversus is present in 20% cases and more than 40% have associated cardiac anomalies [2].

Lee et al. recently reported a 12.1% rate of complex jejuno-intestinal atresias (type IIIb – apple peel atresia and type IV – multiple intestinal atresias) among all patients with small intestinal atresias, and these patients suffered increased morbidity and mortality rates [6]. None of these patients had a combination of type IIIb and type IV [6]. This combination is very rare, and recently was described by Federici et al. [7] and Rich et al. [8].

The association of complex jejunal atresias with apple peel appearance of the remaining ileum along with malrotation and polysplenia in a patient with situs inversus is very rare. There have been only anecdotal reports in literature with similar findings. Rasool et al. reported polysplenia syndrome associated with situs inversus abdominus and type I jejunal atresia in a 2 day old female baby [2]. Abdur Rehman et al. reported a case of dextrogastria, reversed midgut rotation and intestinal atresia in a neonate [9]. However in their case, the spleen was absent. Peetsold et al. reported a girl with apple peel jejunal atresia with situs inverus operated in neonatal period and later presented with duodenal web in adolescence [10]. Ruben et al. have reported a case of discontinuous jejunal atresia with situs inversus without malrotation or splenic malformation [11]. The embryology of situs inversus has not been well established. However the rotational anomaly may result in some vascular accident resulting in multiple atresias and apple peel appearance, as seen in our case.

## 4 Conclusion

The association of situs inversus, polysplenia and complex jejunal atresia is very rare. Pre-operative diagnosis of situs inversus is important for appropriate incision placement and surgical planning.

## Conflicts of interest

Nil.

## Sources of funding

Nil.

## Ethical approval

Ethical approval has been exempted by our institution, SGPGIMS ethics committee.

## Consent

YES, Informed consent was obtained from parents for reporting this case.

## Author’s contribution

Abhishek Chinya: writing the paper.

Kirti Naranje: writing the paper.

Ankur Mandelia: writing the paper, study concept or design, data collection, data analysis or interpretation.

## Registration of research studies

NA.

## Guarantor

Dr. Ankur Mandelia.

## Provenance and peer review

Not commissioned, externally peer-reviewed.
